# Beneficial Effects of Resveratrol on Testicular Functions: Focus on Its Antioxidant Properties

**DOI:** 10.3390/cells14141122

**Published:** 2025-07-21

**Authors:** Adele Chimento, Arianna De Luca, Massimo Venditti, Francesca De Amicis, Vincenzo Pezzi

**Affiliations:** 1 Department of Pharmacy, Health and Nutritional Sciences, University of Calabria, 87036 Rende, Italy; arianna.deluca@unical.it (A.D.L.); francesca.deamicis@unical.it (F.D.A.); vincenzo.pezzi@unical.it (V.P.); 2 Department of Experimental Medicine, University of Campania “Luigi Vanvitelli”, 80138 Napoli, Italy; massimo.venditti@unicampania.it

**Keywords:** male infertility, oxidative stress, reactive oxygen species, antioxidants, resveratrol

## Abstract

Male infertility is a pathological condition that affects many subjects and for which a progressive increase in cases has been observed in recent years. The mechanisms underlying male reproductive system dysfunction are not fully understood and the specific drugs use has not produced optimal results. Therefore, the focus on developing new therapeutic options to prevent or treat this dysfunction is continuously growing. Defective sperm function has been associated with oxidative stress (OS) due to reactive oxygen species (ROS) excessive production. OS is related to mitochondrial dysfunction, lipid peroxidation, DNA damage and fragmentation, and ultimately sperm cell death. Many defense mechanisms to protect from ROS injuries have been developed; natural antioxidants, such as vitamin C and E are able to interact with oxidizing radicals, neutralizing them. Interestingly, resveratrol (RSV), a natural polyphenol with proven health-promoting actions, has been found to be an effective free radical scavenger in several in vitro and in vivo models, providing protection against OS. In this review, we discussed mechanisms related to the modulation of redox homeostasis in the testis and how the alteration of these processes can determine a damage in testicular function; particularly, we focused on the antioxidant properties of RSV that could give beneficial effects in preserving male fertility.

## 1. Introduction

Male infertility is a pathological disorder that contributes to 50% of cases of couple infertility [1,2,3,4]. It is known that some testicular dysfunction, such as hypogonadism, erectile dysfunction, epididymitis, congenital anatomical factors, lifestyle such as tobacco smoking, and obesity, gonadotoxic exposures, and aging, may represent the main causes contributing to male infertility [1,5,6,7]. All these factors are believed to be directly or indirectly involved in the generation of ROS responsible for OS [6]. ROS are a natural bioproduct of oxygen metabolism, and their physiological levels play an important role in almost all crucial phases of sperm function, such as sperm maturation, capacitation, hyperactivation, AR, and sperm–oocyte fusion [8,9]. In human seminal plasma, ROS can originate from exogenous and endogenous sources [10,11]. Excessive alcohol intake, smoking, and environmental factors, such as radiation and toxins, can increase ROS levels in seminal plasma [11]. On the other hand, leukocytes (i.e., neutrophils and macrophages) and immature SPZ represent the endogenous sources of ROS in semen; under normal conditions, leukocytes produce up to 1000 times more ROS than SPZ [12]. Male germ cells can produce limited amounts of ROS [13] which are directly involved in sperm chromatin condensation and in the control of germ cell number through a balance between apoptosis or proliferation [14]. Studies showed how ROS are generated in male germ cells at various stages of differentiation from pachytene SPT to mature caudal epididymal SPZ in rats, mice, hamsters, guinea pigs [13], and in human SPZ at different stages of maturation [15]. In mature SPZ, ROS, through an increase in cAMP-dependent tyrosine phosphorylation of fibrous sheath proteins, lead to SPZ capacitation and hyperactivation [16,17]. Only hyperactivated SPZ proceed to the next stage of the AR. Furthermore, evidence show that ROS increase membrane fluidity and the rate of sperm–oocyte fusion [9].

Testicular germ cells are vulnerable to OS and, in particular, the RS to the LP and the cytotoxic consequences of lipid aldehyde generation [18]; these cells undergo ferroptosis, a cell death dependent on intracellular iron accumulation and peroxidative degradation of lipids and dysfunction of the GSH-dependent antioxidant system [18]. This observation suggests that OS at the spermatid stage underlies the subsequent appearance of functional defects in SPZ, including their competence in sperm–egg recognition [18,19]. ROS overproduction damages SPZ DNA, hindering their fertilization capability. It has been demonstrated that SPZ with defective morphology release greater quantities of ROS compared to SPZ with normal structure [20]. Immature SPZ with altered head morphology and cytoplasmic retention present a higher production of ROS than both mature SPZ and immature germ cells. In fact, while under normal conditions, cytoplasm is extruded from the developing SPZ to prepare for fertilization, a dysregulation of spermiogenesis or spermiation may result in the retention of excess cytoplasm around the midpiece of the damaged SPZ; this residual cytoplasm activates the NADPH system via the hexose-monophosphate shunt, producing ROS [14]. Moreover, sperm function can be affected by increased leukocytes in semen that secrete high amounts of ROS through activation of the mTOR pathway; on the other hand, higher ROS levels upregulate IL-6 via the NFkB pathway in leukocytes [21].

Exogenous sources can also contribute to increased ROS production. Radiation can disrupt the electron flow in the internal membranes of the cells, leading to organelle dysfunction such as mitochondria dysfunction [22,23], while frequent metals exposure, such as cadmium, chromium, lead, mercury, and manganese, causes low sperm quality [24,25,26]. Under normal conditions, enzymatic and non-enzymatic defensive antioxidant systems are present in the seminal plasma, which ensure low concentrations of ROS [27]. Enzymatic antioxidants include GSH reductase, SOD, and CAT, while non-enzymatic antioxidants include vitamins C, E, and carotenoids [27]. When the delicate balance between ROS and antioxidants is perturbed to the point that ROS levels significantly increase or antioxidant levels decrease, OS occurs. Several studies confirmed a link between OS and idiopathic male infertility [28]. Infertile patients have been found to produce a greater quantity of abnormal sperm, which generate more ROS and lower levels of antioxidants, thus leading to OS trough membrane LP, DNA damage, and induction of apoptosis [28,29]. Therefore, tight control of ROS levels during the various stages of spermatogenesis and up to fertilization is of fundamental importance to have optimal sperm parameters necessary for successful conception.

It has been reported that nutritional factors can influence, negatively or positively, sperm quality [30,31,32,33,34,35]; indeed, the observation that healthy dietary models (i.e., Mediterranean diet) [36,37,38,39] as well as an adequate intake of antioxidant molecules [40] are clearly associated with better sperm quality. Natural compounds that possess antioxidant properties can preserve reproductive tissues from several toxic substances. Polyphenols isolated from fruits, vegetables, and edible plants can modulate ROS homeostasis improving male reproductive performance [41,42]. RSV, one of the most investigated natural polyphenolic compound, is contained in several (more than 70) types of plants and in red wine, and ameliorates semen quality in humans, acting as a regulator of male reproductive function [43,44,45]. Several studies confirmed RSV anti-oxidative activity primarily due its ability to modulate antioxidant enzymes expression, such as CAT and SOD [46], which are involved in lipid damage protection of human SPZ during the cryopreservation process [47]. RSV improves sperm motility at low concentrations, while a detrimental effect on this parameter is observed at higher concentrations [48,49]. RSV was able to protect against damage to sperm DNA caused by OS [48] and to ameliorate sperm concentration, total sperm count and motility [50]. In addition, thanks to its scavenger activity, it prevents freezing-induced damage to DNA and lipids during sperm cryopreservation in both infertile patients and fertile men, although it was not able to prevent the decrease in post-freezing motility [47,51]. These effects are dependent on AMPK activation mainly expressed in the post-equatorial region of the sperm head and in the entire flagellum [52]. Illiano and colleagues published a prospective clinical study to evaluate the effects of a RSV-based multivitamin supplement administered daily for 6 months on the sperm parameters of patients with idiopathic infertility [50]. After such treatment, an improvement in sperm motility, total sperm count, and total and progressive motility was observed without any change in sperm morphology, pH, and seminal fluid. Recently, it has been demonstrated that RSV effectively protects the reproductive system against isoflurane-induced toxicity in testicular tissue [53]. Indeed, RSV by inhibiting free radicals and increasing the testicular tissue’s antioxidant capacity, protects sperm parameters, DNA fragmentation, and changes in testicular tissues from isoflurane-induced toxicity [53].

Considering the impact of OS in the male infertility etiology and, at the same time, the importance of natural molecules in counteracting its harmful effects, in this review we have reported current knowledge on the ROS production role in modulating male reproductive function. Moreover, beneficial effects of dietary polyphenols antioxidants properties in male infertility, with particular attention on those exerted by RSV, have been discussed. Understanding the mechanisms underlying the ROS-dependent alteration of male reproductive function and the protective actions of natural polyphenols can provide useful information on both etiology and management of infertility male, helping to identify new therapeutic targets and therapeutic strategies useful for preserving and improving sperm quality.

## 2. ROS Generation and Their Physiological and Pathological Roles in the Testis

ROS are highly reactive chemical species generated during normal oxygen metabolism as bioproducts of cellular respiration within the mitochondrial matrix [54]. They are short-lived molecules containing one or more unpaired electrons (e^−^) in their outermost orbits that tend to stabilize rapidly by donating or accepting e^−^ from other molecules in the environment, and thus acting as reducers or oxidants [55]. The most common types of ROS include O_2_^•−^, HOO^•^, OH^•^ and the ^1^O_2_ [55]. The H_2_O_2_, an important oxidant that participates in the generation of OH^•^, is also considered a ROS [55]. The production of such species can occur not only in the mitochondria but also in the plasma membrane, peroxisomes, the smooth endoplasmic reticulum, and the cytosol, through reactions that can involve various enzymes or transition metals [54]. At low levels, ROS can act as physiological regulators of normal cellular proliferation and differentiation [56]. ROS concentrations are maintained within certain levels in a homeostatic system through enzymatic and non-enzymatic cellular detoxification complexes [54,57]. Enzymatic antioxidant systems include SOD, CAT, GPx, NQO1, HO-1, Trx, and Srx, while non-enzymatic ones include low molecular weight compounds, such as vitamins (vitamins C and E), β-carotene, uric acid, and GSH, a tripeptide (l-γ-glutamyl-l-cysteinyl-l-glycine) that includes a thiol (sulfhydryl) group [54,57]. When ROS levels from both endogenous and exogenous sources increase in the male reproductive tract, the balance between the production of oxidants and the scavenging activity of antioxidant systems is disrupted, thus triggering OS. In fact, the highly reactive nature of ROS means that they can react and modify any molecule, through oxidation, resulting in structural and functional alterations [54]. In particular, the increase in oxygen-derived free radicals alter proteins, enzymes, receptors, and ion channels and causes membrane LP, i.e., the oxidation of fatty acids present in the lipid bilayer which leads to loss of integrity of the plasma membrane and increased passage of ions such as Ca^2+^ or K^+^ by simple diffusion [57]. At the nuclear level, ROS cause oxidative damage to DNA modifying various DNA bases such as cytosine, guanine, and thymine [57], and alter the genes expression involved in the cell death activation such as apoptosis [58] and autophagy [59].

**Figure 1:**
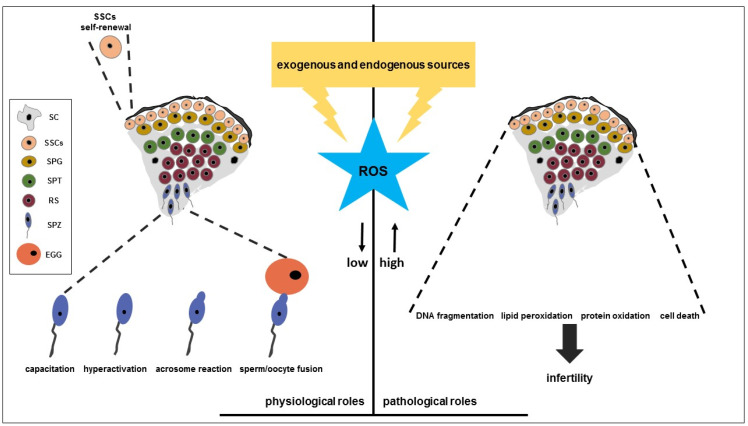
Physiological and pathological roles of ROS in the testis. The drawing represents a cross section of seminiferous tubule, where the spermatogenesis occurs. When ROS levels from both exogenous and endogenous sources are low (↓) they regulate spermatogonial stem cells self-renewal, sperm capacitation and hyperactivation, acrosomal reaction, and sperm–oocyte fusion processes; conversely, in seminiferous tubule the higher (↑) ROS levels cause OS leading to DNA fragmentation, lipid peroxidation, protein oxidation, and consequently cell death in different testicular cell types, resulting in male infertility. EGG: egg cell; SC: Sertoli cells; SSCs: spermatogonial stem cells; SPG: spermatogonia; SPT: spermatocytes; RS: round spermatids; SPZ: spermatozoa.

In the male reproductive system, ROS are involved in the regulation of both physiological and pathological processes (Figure 1); the specific role they play depends on the source, concentration, site of production, and exposure time of the radical species [9,60].

At testicular level, the endogenous ROS sources are primarily SPZ and leukocytes.

Amounts of ROS may arise exogenously from leukocytes that increase in seminal plasma in response to infections or inflammation of the genital tract [61]. Leukocytes, which represent as integral part of the cellular defense system against infections, varicoceles, spinal cord injuries, and inflammation, are able to generate 1000 times more ROS than SPZ [12]. High concentrations of ROS and inflammatory cytokines are strongly correlated with the number of leukocytes in semen collected from sub-fertile men [21]. Their production in activated peroxide-positive leukocytes, including PMN and macrophages, is much higher than that in inactive leukocytes [21]. During chronic infections or inflammations, there is an increase in the cytokines levels in leukocytes that further stimulate ROS production together with a decrease in SOD activity and consequent OS generation [62,63]. Furthermore, seminal plasma, which supports sperm cells, can also represent a ROS source. It contains various enzymes such as NADPH oxidase and xanthine oxidase and molecules like prostaglandins and lipid peroxides contributes to the overall oxidative environment in the male reproductive tract [62]. In addition, several factors including radiation exposure, heat stress, smoking, alcohol use, environmental contaminants, air pollution, and aging represent the ROS exogenous sources; these factors contribute to an increase in the ROS levels beyond a certain threshold limit, damaging testicular functions [64].

In mature SPZ, ROS production occurs endogenously in the plasma membrane by the increased activity of NADPH oxidase or in the inner mitochondrial membrane by the action of nicotinamide adenine dinucleotide NADH-dependent oxidoreductase, following electron leakage from the electron transport chain [61]. In SPZ, elevated ROS production is related to the presence of cytoplasmic droplets or cytoplasmic residues that form in elongated spermatids released from the germinal epithelium; these residues migrate away from the spermatozoon neck towards the end of the mid-tract during transport to the epididymis [65]. In these cytoplasmic droplets, an increased presence of glucose-6-phosphate dehydrogenase has been found, an enzyme that fuels the generation of NADPH which, in turn, by activating NADPH oxidase system, stimulates ROS production [66]. In SPZ, the reactions at the mitochondrial level catalyzed by the NADH-dependent oxidoreductase appear to be the main source of ROS. During spermatogenesis, most of the cytoplasm of SPZ is reduced, confining the mitochondria to the mid-flagellum, whose role is to produce energy in the form of ATP through oxidative phosphorylation, which is necessary for sperm movement [67]. Activation of the mitochondrial electron transport chain via the five protein complexes (complex I–V), which transport electrons from NADPH to O_2_, creates the mitochondrial membrane potential, allowing ATP production [68]. However, a potential loss of electrons from the mitochondrial electron transport chain caused by stress conditions leads to a partial reduction of O_2_ in the radical; following that, SOD will dismutate O_2_^•−^ to H_2_O_2_ in the intermembrane space of mitochondria. Furthermore, O_2_^•−^ reacting with NO is able to generate ONOO^−^ [69]. In addition, high membrane levels of PUFAs, that are highly susceptible to oxidation by free radicals, stimulate the production of highly electrophilic molecules that can disrupt the functionality of the mitochondrial electron transport chain through their covalent binding to mitochondrial proteins, thus dysregulating electron flow [70]. Lipid aldehydes such as acrolein, MDA or 4-HNE, as well as lipid metabolites including lipid peroxyl or alkoxyl radicals can be produced at the mitochondrial membrane [71,72]. A self-repeating cycle is thus created in which the amount of PUFAs is positively correlated with the generation of mitochondrial ROS and lipid aldehydes [72].

Excessive ROS production can cause increased mitochondrial DNA damage, as well as the induction of the mitochondrial apoptotic pathway and, ultimately, a reduction in sperm motility [73,74,75]; indeed, by reducing ROS-induced mitochondrial dysfunction, germ cell apoptosis can be inhibited [67,76]. Mitochondrial LP negatively affects mitochondrial integrity and function, which is one of the main factors underlying the reduction in sperm motility; it causes the loss of MMPs, directly inducing the release of CYTc and thus activating an intrinsic apoptotic pathway. High levels of ROS can oxidize cardiolipin, a phospholipid present in the inner mitochondrial membrane, resulting in the release of CYTc, cleavage of caspases, and increased sperm apoptosis [77,78]. In addition, in response to high intracellular calcium levels, calcium-dependent pores located in the inner mitochondrial membrane of SPZ open, allowing calcium to enter the mitochondria and reducing MMP [79]. A study conducted on infertile men with sperm alterations showed a direct positive correlation between ROS, CYTc release from mitochondria, and the induction of caspases 9 and 3 [73]. Caspases 9 and 3 activation is associated with PARP-1 cleavage and DNA fragmentation increase, leading to poor sperm quality. The MMP decrease and caspase 3 cleavage/activation is also associated with plasma membrane-level exposure to phosphatidylserine in SPZ [75,80,81]. In the control of the first regulatory phase of mitochondria-dependent germ cell apoptosis, changes in the expression levels of pro- and anti-apoptotic proteins of the Bcl-2 family play a key role [82]. A study demonstrated a correlation between excessive ROS production and oxidative damage to mitochondria caused by the liquid storage of goat SPZ and the triggering of intrinsic apoptotic machinery, as confirmed by tandem mass tag (TMT)-based quantitative proteomic analysis that identified specific differentially expressed proteins. In particular, the anti-apoptotic protein Bcl-xL and the proteins NDUFA9, NDUFS2, and NDUFS8, belonging to the subunits of Complex I of the ETC, as well as SDHB, a major subunit of Complex II, were downregulated; conversely, the pro-apoptotic proteins BAX, BAD, and CYTc were upregulated [74].

In addition, AIFM1, a protein released from the mitochondrial intermembrane space during apoptosis, that can induce chromatin condensation and DNA fragmentation through a caspase-independent mechanism, was also significantly upregulated [74]. It has been reported that the PI3K activation leads to the phosphorylation of AKT, which in turn silences the apoptotic pathway, helping to maintain the functionality of SPZ [83]. The PI3K enzyme inhibition and AKT dephosphorylation can promote the intrinsic apoptotic pathway in SPZ, resulting in caspase activation, increased mitochondrial ROS production, oxidative DNA damage, and reduced sperm motility [84]. Furthermore, excessive ROS production can lead to the activation of the MAPK signaling pathway, contributing to changes in the expression of Bcl-2 family proteins and activation of the mitochondrial apoptotic pathway [74,85]. When excessive ROS accumulation occurs, ASK1, a MAPK member, activates the JNK and p38 pathways [86]. The function of this protein is controlled by thioredoxin, the natural inhibitor of oxidant-sensitive ASK1, which under OS cleaves from ASK-1, activating the ASK1 signalosome. This signaling promotes mitochondria-dependent caspases cleavage, triggering the intrinsic apoptosis pathway [87].

ROS can also trigger the extrinsic apoptotic pathway of apoptosis by acting death receptors such as TNF-R1, TRAIL-R1/2, and FAS [87,88]. During death receptor-mediated apoptosis, the adapter protein FADD and procaspase 8 or 10 are recruited to the receptor cytoplasmic surface to form the DISC, which can activate caspase 3, 6, and 7 and initiate germ cell apoptosis [88].

### 2.1. Role of ROS in Modulation of Testicular Physiological Functions

Although excessive generation of ROS could be detrimental, they are required for male reproduction function. Small quantities of ROS regulate spermatogenesis, a physiological process where, starting from SSCs, through mitotic and meiotic divisions, SPG, SPT, and spermatids originate and differentiate into SPZ through a series of morphological changes known as spermiogenesis [89]. The spermatogonial maintenance and differentiation are controlled not only by growth and transcriptional factors but also by physiological levels of ROS [90,91] (Figure 1). It has been shown that the GDNF and FGF2, secreted by SC, activate RTK receptors and induce a signaling cascade that drives SSCs self renewal [90,91]. In SSCs, the GDNF- and FGF2-dependent pathways activation cause transcriptional mRNA increase of BCL6B, a factor involved in NOX1 gene expression regulation; the latter codes for an enzyme involved in the ROS generation which is involved in the MAPKs phosphorylation [91]. In these cells, a positive feedback mechanism has been proposed in which ROS produced by NOX1 phosphorylate and activate MAPK14, which in turn downstream phosphorylates and activates MAPK7. MAPK7 targets BCL6B which initiates ROS production by increasing NOX1 expression, driving mouse SSCs self-renewal [91]. In the testis, ROS at low level can regulate SPZ capacitation, hyperactivation, AR, and sperm–oocyte fusion (Figure 1) [92].

Capacitation is a process that provides SPZ a series of properties essential for achieving fertilization, including increased motility, recognition of the oocyte’s zona pellucida by the spermatozoon, and acrosomal exocytosis. Although the molecular mechanisms underlying capacitation have not been fully defined, it is known that it involves an efflux of cholesterol from the spermatozoon’s plasma membrane and an increase in tyrosine phosphorylation [16]. The removal of cholesterol is necessary to increase membrane fluidity and permeability, preparing the sperm for the AR and helping it to penetrate the outer layer of the egg [93]. Moreover, the activation of specific signal transduction pathways that regulate tyrosine phosphorylation represent a key event responsible for increasing the sperm membrane fluidity, thus preparing it for the AR and fertilization [94,95]. In recent years, several studies confirmed some past intuitions [96,97,98] on the role of low levels of exposure to physiological ROS to drive the signal transduction processes associated with sperm capacitation [16,99]. ROS generation is thought to exert a positive influence on tyrosine phosphorylation, as demonstrated in the SPZ of humans [100], rats [101], mice [102], bulls [103], horses [104], and boars [105]. At the molecular level, the mechanisms by which ROS stimulate sperm capacitation involve the stimulation of adenylate cyclase activity [106], accompanied by the activation of protein kinase A [107,108]; the induction of cholesterol oxidation and subsequent efflux from the plasma membrane [109]; the activation of ERK-like pathways [110]; and the inhibition of tyrosine phosphatase activity [107]. In particular, it has been demonstrated that at the level of SPZ, the O_2_^•−^ produced by NADPH oxidase at the plasma membrane or at the mitochondrial level during steady-state respiration, combines with NO produced by NOS, giving rise to the powerful oxidant ONOO^−^, which mediates the oxidation of cholesterol to oxysterols [16]. These oxysterols significantly increase the fluidity of the membrane [93]. The combined action of ONOO^−^ and H_2_O_2_ in turn, generated by the action of SOD, simultaneously determines the inhibition of the activity of tyrosine phosphatase [94]. Furthermore, the O_2_^•−^ in the presence of Ca^2+^ activates soluble adenylyl cyclase, thus stimulating the production of cAMP and the activation of PKA [16,106], which is involved in the SRC activity, increase [111]. However, both the PKA activation and the serine–threonine phosphatase down-regulation by SFK are required for the human SPZ functional capacitation [16,107,111]. In addition, it has been observed that capacitation in vivo is also associated with an increase in thiols on some proteins of the spermatozoon surface as a consequence of OS through the generation of NADPH, fueled by the hexose monophosphate shunt [112]. The presence of such groups would allow the release of fully capacitated cells on the surface of the oviductal epithelium [112]. The achievement of full capacitation involves hyperactivation of sperm motility and preparation for the AR, an essential step for the penetration of the sperm into the oocyte (Figure 1) [113]. Hyperactivation is the condition of sperm motility characterized by increased and asymmetric flagellar movement, high lateral displacement of the head, and non-linear motility of the sperm [114]. Also for this particular condition, physiological levels of ROS have a positive impact [115]. There are multiple molecular mechanisms underlying the initiation process of hyperactivation [114] that include cytosol alkalinization and increased influx of Ca^2+^ and HCO_3_^−^. The increase of Ca^2+^ depends primarily on the inactivation of an ATP-dependent Ca^2+^ regulatory channel (plasma membrane Ca^2+^-ATPase, PMCA), a pump that extrudes Ca^2+^ from the cell’s cytoplasm and into the extracellular space, thereby regulating intracellular Ca^2+^ concentration [116]. The regulation of the homeostasis of HCO_3_^−^, which together with the removal of H^+^ from the cytoplasm contributes to the alkalization of intracellular pH, is influenced by a wide variety of transporters, exchangers, and enzymes [117]. Among these proteins are the CAs, metalloid-enzymes that catalyze the hydration of CO_2_ into HCO_3_^−^ [118], the cotransporters such as the Na^+^/HCO_3_^−^ (NBC), and the solute carrier SLC4 A1 (AE1), which transport the substrates HCO_3_^−^ and Cl^−^ [119] in order to maintain the HCO_3_^−^ concentration during capacitation. Calcium and HCO_3_^−^ ions modulate the activity of the sAC, leading to the production of cAMP that, by activating PKA, triggers NADPH oxidase such as NOX5 and stimulates increased ROS generation [119]. PKA is also responsible for the phosphorylation of serine and tyrosine residues by a PTK which in turn phosphorylates tyrosine residues in the fibrous sheath around the axoneme and cytoskeleton of the sperm flagellum, resulting in increased motility [119]. The H_2_O_2_ generation causes increased tyrosine phosphorylation by both inducing PTK and inhibiting PTPase, the enzyme that determines the dephosphorylation of tyrosine residues [119]. It has also been reported that ROS modulate other crucial processes involved in the attainment of sperm fertilizing ability such as AR and sperm–oocyte fusion (Figure 1) [92]. The AR, a well-controlled exocytosis process that occurs after capacitation, is a crucial event for the fertilization of mature oocytes. It involves the activation of several protein kinases such as PKA, PKC, and PTK that regulate the secretion of the acrosomal matrix rich in digestive enzymes (e.g., acrosin and hyaluronidase) that allow the penetration of SPZ through the cumulus cells and zona pellucida [120]. These protein kinases may be involved in the regulation of intracellular Ca^2+^ not only during capacitation but also in the AR [120]. In particular, the AR is facilitated by protein tyrosine phosphorylation and Ca^2+^ influx; the latter causes an increase in intracellular cAMP and PKA, which can activate protein tyrosine phosphorylation through various mechanisms leading to increased actin polymerization, a process essential for increased motility. Moreover, the influx of Ca^2+^ during capacitation causes the cleavage of PIP2 into DAG and IP3 [120]. IP3, by activating actin-separating proteins, induces the fusion of the acrosomal and plasma membranes, ultimately determining acrosomal exocytosis. DAG, by activating PKC, determines a further Ca^2+^ influx and activation of PLA2, which catalyzes the cleavage of secondary fatty acids from the triacylglycerol backbone of membrane phospholipids; this ultimately results in increased fluidity of the sperm plasma membrane, essential for correct sperm–oocyte fusion [120]. In this context, it has been observed that ROS facilitate AR. Studies have, in fact, demonstrated their role in the PKA activation and proteins tyrosine phosphorylation as well as in PKC activation, resulting in higher enzymatic PLA2 activity [121,122]. Moreover, the NO through the synthesis of the second messenger cGMP and the activation of kinases (PKC and PKG) [123], regulates SPZ membrane fluidity during AR.

### 2.2. Role of ROS as Mediators of Pathological Effects in the Testis

High levels of ROS can damage germ cells at various stages of development, particularly primary and secondary SPT and SPZ, while early germ cells such as SPG are more resistant than late stage ones (Figure 1) [124]. The amount of ROS that comes from exogenous sources such as radiation exposure, heat stress, environmental contaminants, smoking, alcohol use, air pollution, and aging, unbalances oxidative homeostasis and contributes to OS, damaging testicular functions [64,125]. Evidence suggested that radiation toxicity in the testis depends on both its thermal and non-thermal effects. Thermal action is related to their ability to increase scrotal temperature, disrupting cell function and promoting testicular germinative tissue loss and spermatogenetic arrest in humans [126]. Non-thermal actions of radiation include increased levels of seminal ROS and reduced antioxidant enzymes, associated with chromosomal abnormalities, micronuclei formation, sperm membrane potential alterations, and apoptosis induction [127]. Data suggested that DEHP, a plasticizer used in the production of paints, food packaging, medical devices, and children’s toys, has been shown to have toxic effects by inducing OS in the testis [128]. Its exposure affected male reproductive function by ROS increase and antioxidant systems reduction such as GPx and SOD [128,129]. Smoking and alcohol are both exogenous factors that cause an imbalance between ROS production and antioxidant defense mechanisms in the sperm. Smoking can increase the leukocytes number ROS seminal levels as well as decrease the seminal plasma antioxidant systems [130,131]. Excessive alcohol consumption impairs both the quantity and quality of sperm parameters [132,133]. Ethanol alters the structure and function of mitochondria and reduces respiration and ATP levels by increasing the production of ROS through its metabolism in the liver [134]. It also increases the activities of cytochrome P450 (CYP2 E) enzymes, which in turn increase the activity of NADPH oxidase and the production of superoxide anions [134]. Moreover, an increase in ROS levels may also depend on the accumulation of acetaldehyde, a by-product of ethanol metabolism that can interact with proteins and lipids, thus damaging cellular components and decreasing the percentage of normal SPZ [135]. Exposure to environmental contaminants such as cadmium, mercury, bisphenol A, and dioxin by increasing ROS levels and triggering redox-sensitive pathways deteriorate sperm parameters, the integrity of Leydig and Sertoli cell function, hormone biosynthesis and gene expression, and epigenetic modifications. Chemical components of air pollution also negatively affect reproduction, causing OS, inducing LP, and enhancing the binding of PAHs to their receptors, or binding to PAHs to cause DNA strand breaks [136]. Furthermore, increased ROS production in the testis may play a role in the age-related degeneration processes associated with male infertility. In aged humans, excessive generation of endogenous ROS and decreased activity of antioxidant enzymes damage Leydig cells. It has been reported that both FSH and hCG, by stimulating cellular metabolism, produce ROS that influence the differentiation processes in germ cells. Furthermore, as a result of ROS increase, the activities of several P450 enzymes that regulate testicular steroidogenesis in Leydig cells are reduced, resulting in decreased T synthesis and secretion [64].

The vulnerability of SPZ to OS is mainly due to the high content of PUFAs in their membranes, the limited availability of antioxidant enzymes and the lack of effective DNA repair mechanisms. OS leads to harmful molecular changes, including DNA fragmentation, LP, and protein oxidation (Figure 1). In addition, excessive levels of ROS can trigger cell death mechanisms including apoptosis. Although sperm DNA is highly compacted and associated with protamine that confer some protection against external damage [137] and repair mechanisms are triggered in case of damage [138], it is vulnerable to OS [139]. The OS damages sperm DNA by fragmenting it, promoting the formation of single- and double-strand breaks and generating oxidative base adducts. The formation of 8-OHdG, one of the most documented oxidative DNA adducts resulting from guanine oxidation underlying chromosomal instability and mutagenesis, serves, in fact, as a biomarker of oxidative stress in SPZ [140]. While ROS-mediated single-strand breaks can be repaired by the oocyte after fertilization, double-strand breaks are more difficult to repair and are often associated with severe chromosomal damage [141]. SPZ with fragmented DNA have a lower fertilization capacity because DNA damage compromises the ability of the sperm to efficiently transfer the paternal genome to the oocyte, increasing the likelihood of implantation failure, spontaneous abortion, and development of genetic abnormalities in the offspring [141]. Furthermore, the availability of SPZ of only one active enzyme for DNA repair, OGG1, responsible for the repair of oxidative base lesions, makes them highly susceptible to the accumulation of DNA damage caused by OS [142]. The presence in the sperm membrane of PUFAs with multiple double bonds makes the SPZ particularly vulnerable to OS with generation of LP [143]; this event is characterized by the formation of lipid peroxides and various toxic by-products that trigger a cascade of cellular damage, compromising SPZ functionality. In particular, ROS remove a hydrogen atom from the methylene group of a PUFAs, creating a lipid radical that reacts with molecular oxygen to cause the successive formation of lipid peroxyl radicals, lipid hydroperoxides, and highly toxic by-products such as MDA, acrolein, and 4-HNE, which can further damage membrane lipids, proteins, and even DNA [143]. Since the PUFAs-rich sperm membrane ensures membrane fluidity, essential for sperm motility and the ability to undergo capacitation, the impact of LP on SPZ function is considerable. Furthermore, LP, by damaging the acrosomal membrane responsible for the release of hydrolytic enzymes during the AR, prevents the sperm from binding to the egg and fertilizing it [144]. ROS can interact with specific amino acids, such as cysteine, methionine, and tyrosine, which contain sulfur or aromatic groups that are highly susceptible to oxidation, thus causing protein carbonyls and disulfide bridges formation, which alter the sperm proteins structure and function [145]. Such oxidative modifications cause the aggregation of key proteins involved in sperm motility, structure, and fertilization, further contributing to male infertility. In SPZ, one of the main targets of protein oxidation are the cytoskeletal proteins actin and tubulin which are structural components of the sperm flagellum [146]. In addition, the enzymes involved in sperm metabolism and energy production are also affected by OS [146]. Studies have shown that creatine kinase and adenylate kinase, which play a role in the ATP generation necessary for sperm motility and AR, can undergo oxidation, resulting in a reduction in their activity [147]. Oxidative damage to proteins involved in the AR, such as acrosin [148], also compromises the ability of the sperm to release these enzymes, preventing penetration into the oocyte. Studies confirmed that infertile men showed a significant BAX increase and BCL2 decrease in seminal fluid at both mRNA and protein levels [149]; moreover, the mature SPZ from infertile patients with increased ROS levels had significantly higher levels of apoptosis than mature SPZ from the control group [150]. These observations suggested a close correlation between OS, apoptosis induction, and infertility (Figure 1). Among the various germ cell types, only the more advanced stages of germ cell development, such as spermatids and SPZ, but not SPG, showed apoptosis along with severe oxidative damage after Hx/XO treatment [124]. The tolerance of SPG to OS is due to the presence of high levels of Cu/Zn SOD and Zn, which protects it from ROS damage [124]. More recent studies have confirmed that ROS-dependent epigenetic modulations are implicated in male germline apoptosis [151]. The OS, determining modifications such as DNA hypermethylation, histone acetylation, chromatin remodeling, and the transition from histones to protamines, causes alterations in the expression of specific genes involved in the induction of apoptosis of male germ cells [151].

To better understand how changes in ROS levels affect sperm function and fertilization, genetic mouse models have been created by deleting or overexpressing genes involved in ROS production or scavenging. The available knockout (KO) mouse models helped to better understand the role of different antioxidant enzymes in male fertility. A study, demonstrated the role of GSS/GSH in male germ cells though conditional deletion of Gss mice via Stra8-Cre (S8) [152]. The authors showed that 8-month-old S8/Gss−ss−/− male mice exhibited significantly reduced fertility, confirming how in germ cells GSS plays a crucial role in the resistance to OS injury in aged mice. Furthermore, the study has deepened the understanding of ferroptosis during spermatogenesis and suggested that the inhibition of this cell death type may be a potential strategy for male infertility treatment. Alterated levels of TMEM225, a testis-specific protein which is essential for proper sperm maturation in the epididymis, have been found in patients with nonobstructive azoospermia, suggesting its role in male fertility. A Tmem225 KO mouse model, obtained by using the CRISPR/Cas9 system, has been generated to explore its function and mechanism in male reproduction. Male Tmem225 KO mice were infertile and SPZ lacking TMEM225 exhibited mitochondrial dysfunction, impaired glycolysis, high ROS levels, reduced motility, and flagellar folding, leading to typical asthenospermia [153]. Moreover, the KO mice for Prx4, a protein with thioredoxin-dependent peroxidase activity, also reported to have testicular atrophy, elevated OS, and cell death in spermatogenic cells, suggesting its importance in male reproduction [154]. In addition, to determine the role of CAT in mitigating the testicular redox dysfunction observed with aging, transgenic CAT overexpressing mice (MCAT) were created. These animals showed no age-dependent loss of testicular germ and SC, or SPZ compared to wild-type mice. Low overall ROS and reduced ONOO^−^ levels were found in SPT of aged MCAT mice following exposure to the prooxidant tert-butyl hydroperoxide. Furthermore, 8-OHdG OS-dependent DNA lesions were also significantly reduced in aged MCAT mice [155].

## 3. Antioxidant Properties of RSV and Health Benefits

RSV (3,5,4′-trihydroxystilbene) is a natural nonflavonoid polyphenol found in numerous plant species, particularly in grapes, blueberries, and peanuts; it exerts multiple beneficial effects on human health in a variety of organs and systems related to its anti-inflammatory, cardioprotective, antitumor, neuroprotective, and antioxidant properties [43,156,157,158]. The antioxidant capacity of RSV is strongly dependent on the redox characteristics of the phenolic OH groups and the possibility of electron delocalization at the level of its chemical structure making it more effective in the interaction with free radicals that are transformed into more stable molecules [46,159]. The protection from the damaging effects of OS by RSV may not only be due to its direct free radical scavenger action but also to other mechanisms including (a) enhancing endogenous antioxidant enzymes; (b) promoting antioxidant molecules and the expression of related genes involved in mitochondrial energy biogenesis, mainly through the AMPK/SIRT1/NRF2 [160,161], ERK/p38 MAPK [162], and PTEN/AKT signaling pathways [163]; and (c) inducing autophagy via the mTOR-dependent or TFEB-dependent pathways [164]. Indeed, RSV has been reported to inhibit the activity and expression of NADPH oxidase, as well as upregulate tetrahydrobiopterin synthase guanosine triphosphate cyclohydrolase I and reduce endothelial NO synthase. In vitro studies confirmed the scavenger potential of RSV [165] and its ability to influence the regulation of redox systems by directly neutralizing different ROS, such as OH^•^, H_2_O_2_ and ONOO^−^, and NO [46,166]. RSV stimulate antioxidant defense systems and increase the expression of various antioxidant defensive enzymes such as HO-1, CAT, GPx, and SOD. RSV, moreover, induces GSH level responsible for maintaining the cellular redox balance, and these actions depend on the regulation of various signaling pathways including NRF2, SIRT1, and NFkb [167]. At the molecular level, RSV increases the translocation of NRF2 into the nucleus by disrupting NRF2-KEAP1 binding; the NRF2-KEAP1 dissociation is facilitated by RSV p62-NRF2 complex formation. Moreover, it also activates NRF2/ARE through the stimulation of p38 MAPK and SIRT1/FOXO1 pathways. The RSV-mediated NRF2 expression increase is dependent on the suppression of the AKT/ERK1/2 inhibitory signaling pathway [168]. Then, NRF2 translocating to the nucleus binds to the ARE, initiating the transcription of many antioxidant genes such as SOD and CAT [168]. RSV promotes the transcriptional functions of FOXOs in the nucleus to facilitate the transcription of many antioxidative genes contributing to the reduction in OS [46]. Moreover, it upregulated PTEN, a major antagonist of PI3K, blocking the AKT activation. Consequently, the activated AKT reduces, leading to decreased FOXOs phosphorylation. Therefore, less phospho-FOXOs translocate from the nucleus to the cytoplasm, and more FOXOs remain in the nucleus to act as transcriptional factors [46]. Evidences confirmed that RSV activates AMPK to maintain the structural stability of FOXOs, facilitate their translocation, and accomplish their function [46]. In addition, the activated AMPK phosphorylates PGC-1α, which can translocate into the nucleus and can be deacetylated by SIRT1 [46,169]. Then PGC-1α promotes NRF2, leading to increased anti-oxidative gene expression and then reduced OS [169]. Autophagy induction underlies the protective effects of RSV; this event occurs by activating TFEB, which promotes the formation of autophagosome and their fusion into an autolysosome, leading to reduced OS [164]. RSV protective actions against OS are also attributable to its ability to suppress H_2_O_2_-induced activation of NFkB, a factor involved in the regulation of genes during inflammation and apoptosis [170]. Moreover, this polyphenol is able to reduce H_2_O_2_-mediated apoptosis by positively regulating the expression of cleaved caspases 3 and 9 and reducing the ratio of BAX to BCL2 [171].

Growing evidences demonstrated that testicular steroidogenesis dysfunction triggered by OS is related to reduced male fertility [172]. A strong correlation between steroidogenesis disruption and LP has been reported, and it has also been shown that reduced testicular steroidogenesis under OS occurs through the inhibition of key transcription factors regulating steroidogenic enzyme genes expression [172]. Several factors could contribute to OS-dependent male infertility such as pesticides, heavy metals, industrial chemicals, obesity, tobacco and alcohol consumption [125,173], and CAF (cafeteria) diet [174]. Furthermore, pathological causes of male infertility are also known and can be subcategorized as testicular, pre-testicular, extra-testicular, and idiopathic infertility. The latter, which comprises 30–40% of cases, is caused by endogenous and exogenous factors that induce OS [175]. In recent years, an increasing interest has been directed to the use of natural supplements as therapeutic or protective approaches for male fertility disorders [174]. Several in vitro and in vivo studies have confirmed RSV antioxidant actions at the testicular level [45,176]; furthermore, a recent retrospective pilot clinical study confirmed the positive impact of RSV-based nutraceutical combined with surgical scleroembolization in reducing the time needed to fully recover SPZ function [177]. RSV exerts a protective action at the testicular level by acting as a free radical scavenger and preventing LP, an event that alters the fluidity and permeability of the sperm membrane, reducing both sperm motility and the ability to fertilize the oocyte [44,178]. In SPZ, RSV, by modulating various signaling pathways such as AMPK, improves mitochondrial functions and, consequently, enhances the antioxidant defense system, determining the balance of ROS and a better sperm quality [44]. In addition, the proven antioxidant activities of RSV suggest it as an effective compound in preventing sperm damage induced by freezing during cryopreservation [49]. Several studies in animal models have demonstrated that in the testis, RSV, by reducing OS, prevents the apoptotic pathways activation triggered by exposure to environmental contaminants (i.g. sulfoxaflor) [179], drugs (i.g. MTX, cisplatin) [180,181], or initiated in pathological conditions such as diabetes [182] or varicocele [183]. Particularly, in an experimental rat model with varicocele, it has been showed that RSV exerts its protective effects by reducing OS-mediated apoptosis and inflammation, as confirmed by gene expression reduction in ASC, NLRP3, caspase 1 and BAX levels, and increase in those of BCL2 [183].

## 4. Antioxidant Activity of RSV in the Testis: In Vitro Studies

The antioxidant actions of RSV in the various types of testicular cells have been confirmed by several in vitro studies (Table 1). It has been reported that OS causes LP of Leydig cells, lipoprotein damage, misfolded proteins, and DNA fragmentation contributing to male infertility, and that the bioactive components of medicinal plants could improve Leydig cells functionality [184]. ROS can also induce a negative impact on Leydig cell steroidogenesis and the p38 MAPK is a kinase involved in ROS-induced steroidogenic dysfunction [185]. Banerjee and colleagues demonstrated that RSV exerted a protective effect on B(a)P-induced steroidogenesis dysfunction and StAR gene expression in both TM3 immortalized mouse Leydig cell line and primary Leydig cells isolated from 8-week-old adult male Wistar rats; these animals received RSV (50 mg/kg/day) alone or in combination with B(a)P (5 mg/kg) for 60 days. Results evidenced that, while B(a)P caused OS, RSV was able to significantly prevent B(a)P-induced ROS generation in both cell models; moreover, in isolated Leydig cells it increased the gene expression of antioxidant enzymes such as SOD1, SOD2, CAT, and GPx which was decreased by B(a)P [186]. In addition, it also prevented the activation of stress kinases such as p38 MAPK and increased StAR protein levels by reducing DAX-1 expression and increasing that of SF-1 [186]. Another study confirmed that in TM3 cells, the H_2_O_2_-damages were ameliorated using RSV which significantly increased their metabolic activity and cell membrane integrity concomitantly with a decrease in O_2_^•−^ [187]. The protective effects on the structural and functional integrity of TM3 cells in the case of H_2_O_2_-induced OS were confirmed by the same authors in another study [188]. It was observed that low dose of RSV (10µM) in both normal culture conditions and after OS, increased the TAC and improved intercellular communication by increasing GJIC [188]. It has been shown that there is in fact a direct link between OS and gap junctions alteration, with some studies evidencing as many nutraceuticals and antioxidants are able to repair the GJIC [189]. TM3 cells were used to study the RSV protective effects on nicotine-induced OS [190]; the significant antioxidant actions were confirmed by both the reduction of ROS and MDA levels and increase of those SOD in cells treated with different RSV doses (2–10–50 µM) [190]. In 2022, Xu and co-workers demonstrated that in TM4 immortalized mouse Sertoli cell line, RSV protected against OS and apoptosis induced by the mycotoxin ZEN through PI3K/AKT-mediated activation of the NRF2/HO-1 signaling pathway. In particular, RSV was able to increase CAT activity and GSH and decrease MDA and ROS levels. It also upregulated AKT phosphorylation, NRF2 nuclear translocation, and HO-1 expression under OS conditions [191]. RSV (30 µM) counteracted B(a)P-dependent reproductive toxicity and of its active metabolite benzo(a)pyrene-7, 8-dihydrodiol-9, and 10-epoxide (BPDE) also in GC-2 immortalized cells derived from mouse SPT. In fact, these two endocrine disruptors induced mitochondrial damage through the ROS production which suppresses SIRT1/TERT/PGC-1α signaling; RSV was able to increase the activity and expression of SIRT1 attenuating these harmful effects [192]. Studies focused on RSV protective effects evaluation on bovine SPZ exposed to OS using ferrous ascorbate also confirmed that its supplementation (10–25–50 μM) preserved SPZ vitality. This effect was associated with a significant increase in intracellular SOD activity and GSH concentrations [193]. Moreover, this natural compound was able to reduce ferrous iron/ascorbate-dependent ROS and LP generation in mouse SPZ, and preserving the in vitro fertilization capability [194], as confirmed by other studies [195]. It is known that cryopreservation process causes in SPZ several stress types such as thermal shock and osmotic stress induced by ice crystals causing physical and chemical damage, including SPZ plasma membrane deterioration and alteration of their functionality [196]. In addition, the freezing and thawing process causes OS and consequent reduction in sperm motility, viability, and DNA integrity [196]. RSV effects on the quality and redox status of cryopreserved bovine semen was evaluated by Correa and colleagues [197]. In particular, they demonstrated that RSV significantly increased TAC levels and reduced ROS production of cryopreserved semen when used at 50 and 20 μM, respectively; however, its use between 10 and 50 μM decreased post-thawing semen quality parameters [197]. Recently, in another study the in vitro effects of two different concentrations (12 and 30 µM) of RSV were evaluated on motility of SPZ from 154 subjects with AZS [198]. It has been observed that after 1 h at 37 °C, the control group and the one treated with the lowest dose of RSV presented a slight increase in progressive motility (PM) and a simultaneous decrease in non-progressive motility (NP); in the group treated with the highest dose of 30 µM of RSV, all motility parameters decreased significantly. Furthermore, a significant decrease in ROS levels was observed with both RSV concentrations, including the one that impaired sperm motility. These results suggested that an excessive OS reduction at the highest doses of antioxidants could cause a redox imbalance that would paradoxically worsen the seminal parameters of subjects with AZS [198].

**Table 1:** Antioxidant effects of RSV in the testis: in vitro studies. The symbols indicate increase (↑) or decrease (↓).

Testicular Cell Type	Experimental Model	RSV Dose	Antioxidant Mechanisms	Reference
Leydig	TM3	10 µM	ROS ↓	[186]
Leydig	TM3	10 µM	O_2_^•−^ ↓	[187]
Leydig	TM3	10 µM	TAC ↑	[188]
Leydig	TM3	2–10–50 µM	MDA ↓ ROS ↓ SOD ↑	[190]
Sertoli	TM4	2.5 µM	CAT, GSH ↑ NRF2/HO-1 signaling pathway ↑ MDA ↓ ROS ↓	[191]
Spermatocytes	GC-2	30 µM	ROS ↓ SIRT1/TERT/PGC-1 a signaling ↑	[192]
Spermatozoa	Bovine	10–25–50 µM	SOD, GSH ↑	[193]
Spermatozoa	Mouse	15 µM	ROS ↓ LP ↓	[194]
Spermatozoa	Bovine	20–50 µM	ROS ↓ TAC ↑	[197]
Spermatozoa	Human	12 µM	ROS ↓	[198]

## 5. Antioxidant Activity of RSV in the Testis: In Vivo Studies

RSV beneficial effects related to its antioxidant actions have been demonstrated mostly in in vivo studies (Table 2). By feeding sexually mature Duroc boars a normal diet and an RSV-based diet of 20 mg/kg/day for 14 days, the beneficial effects of RSV on spermatogenesis were investigated [199]. Semen analysis showed that volume, density, motility, and viability of SPZ of breeding boars fed with RSV were significantly higher than those of the control group, while the rate of sperm malformations was significantly reduced. RSV beneficial actions on sperm quality correlated with a significant increase in the antioxidant activity of SOD and a decrease in plasma levels of MDA which confirmed its ability to ameliorate genital lesions caused by oxidation [199]. In addition, it promoted spermatogenic cells division and differentiation, as evident from the tissue analysis, and increased GnRH, FSH, LH, and T serum levels; these results confirmed its ability to improve reproductive function by strengthening the hypothalamic–pituitary–testicular axis activity [199].

Several chronic diseases, such as diabetes, can alter normal testicular function by suppressing sperm count, motility, and vitality and increasing sperm abnormalities [200]. The deleterious alterations in testicular tissue architecture associated with this pathology are related to OS induction dependent on antioxidant enzyme activity inhibition, ROS and LP increase, as well as to the apoptosis initiation. RSV administration (50 mg/kg/day for 4 consecutive weeks) in STZ-diabetic adult male Wistar rats ameliorated diabetes-induced testicular damage [182]. These beneficial actions were dependent on its ability to decrease H_2_O_2_ production and increase of SOD, CAT, GPx and GSH activities; furthermore, it triggered antiapoptotic mechanisms as confirmed by BCL2 expression increase and by reduction in BAX levels, CYTc release, and caspase 9 and 3 cleavage after RSV use [182]. Simas and colleagues confirmed the RSV ability to improve sperm DNA quality and reproductive capacity in type 1 diabetes, independently of insulin therapy [201]. Particularly, RSV (150 mg/kg/day from 33 to 110 days postpartum) administrated as adjuvant in the STZ-diabetic male Wistar rats, activated specific reactions against hyperglycemia by reducing testicular and epidydimal levels of MDA, a marker of LP, and then controlling the OS [201]. In another study, RSV administration (150 mg/kg/day for 21 days) to STZ-diabetic adult male rats attenuated the abnormal reproductive parameters, restored the antioxidant mechanism by increasing the activities of SOD, CAT, and GPx and reducing the MDA levels; these effects were also accompanied by reduction in inflammatory responses (TNF-α, IL-6, IL-4, and IL-10 inhibition) and improved insulin resistance [202].

The intensive use of pesticides in agriculture is not only a cause of environmental pollution, but also determines their passage along the food chain with consequent accumulation in animal tissues with harmful effects on human health [203]. In particular, environmental pollutants by acting as endocrine disruptors can inhibit male reproductive functions, altering the expression of genes related to the spermatogenesis and steroidogenesis regulation, inducing ROS production or damaging BTB integrity [203]. The negative impact on the male reproductive system of GLF, the most commonly used pesticides, and the protective effects of RSV were evaluated in male albino Wistar rats fed a normal diet, or containing only RSV (20 mg/kg/day) or GLF (375 mg/kg/day) for 8 weeks and others with one containing both GLF and RSV [204]. The results obtained showed that GLF administration reduced sperm motility, sperm plasma membrane integrity, GSH and SOD levels in rat testicular tissue; RSV use in the diet protected spermatological parameters and DNA damage, reducing GLF-induced MDA levels, improving the antioxidant defense mechanism (GSH and SOD) and regenerating tissue damage in rats testis [204]. Jalili and co-workers showed that RSV administration (2, 8, 20 mg/kg daily for 65 days) in male Wistar rats alongside malathion, an organophosphate compound widely used in agricultural fields and gardens to destroy parasites, considerably counteracts its testicular toxicity [205]. Results showed that sperm parameters, such as morphology, viability and count, as well as T levels and germinal layer height, were improved by all the RSV doses used. Interestingly, these effects were related to an RSV-dependent increase in TAC and a reduction in testicular MDA levels and LP [205]. The protective effect of RSV on synthetic plastic polymer PVC-induced reproductive toxicity was also evaluated in adult male Wistar rats [206]. RSV (20 mg/kg/day for 60 days) administration together with that of PVC, determined a significant increase in reproductive organ weight, sperm count, viable and motile SPZ, daily testicular SPZ production, steroidogenic enzyme activities and serum T levels compared to those treated with PVC alone [206]. In addition, in the same group, LP decreased along with an increase in antioxidant enzyme activity levels (i.e., SOD, CAT), thus confirming that RSV use contributed to the improvement of steroidogenesis, spermatogenesis, and OS mitigation [206]. The same RSV protective effects were observed in insecticide sulfoxaflor-induced testicular toxicity in adult male Sprague Dawley rats [179]. The RSV (20 mg/kg/day for 28 days) co-treatment reduced testicular MDA, GSSG and NO levels, while increasing GSH content compared to sulfoxaflor-treated rats; in addition, it suppressed apoptosis, reduced testicular DNA fragmentation and seminiferous tubule degeneration, thus improving the overall spermatogenesis process [179].

The integration of engineered nanomaterials in nanotechnology applications has achieved remarkable results in various fields; in particular, nanosized iron oxide (Fe_2_O_3_-NP) is one of the most widely used engineered nanomaterials in coating products, plasters, clay modeling, and metal surface therapy products [207]. Furthermore, its use in the induction of magnetic hyperthermia for cancer treatment, or as a carrier for targeted drug delivery or for tissue repair via welding or soldering, is also known [207]. However, it can exert several toxic effects through the induction of OS [207]. The efficacy of RSV (20 mg/kg once daily for 8 weeks in adult male Wistar albino rats) in alleviating impaired sperm quality and testicular damage resulting from exposure to nanosized iron oxide (Fe_2_O_3_-NP) was confirmed in another in vivo study [208]. In particular, the detrimental effects induced by Fe_2_O_3_-NP on sperm motility and viability were significantly counteracted by RSV administration. It also restored other Fe_2_O_3_-NP-dependent effects including the depletion of T, FSH, and LH, testicular expression levels of steroidogenesis-related genes (3β-HSD, 17β-HSD and NR5A1) as well as SOD, CAT, GPx, and GSH, while reducing MDA levels [208].

Studies showed that long-term/chronic use of drugs such as morphine [209] or exposure to waste anesthetic gases can damage the genome and lead to OS [210]. A study conducted in male BALB/c mice demonstrated that RSV (2, 8, 20 mg/kg/day for 2 weeks) administration due to its antioxidant properties can improve sperm quality and prevent morphine-induced adverse effects on sperm parameters [211]. The results indicated that RSV attenuated the harmful effects of morphine by increasing T levels, sperm count, vitality and motility, and testicular weight, and reducing NO levels compared to the group of animals treated with the drug alone [211]. Isoflurane, an inhalation anesthetic widely used for general anesthesia in animals and humans, adversely affects the reproductive system of humans and experimental animals by altering sperm parameters [212]. Although the mechanism by which it causes these effects is unclear, it is thought to interfere with tissue antioxidant defenses by altering the balance of free radicals. A study conducted using adult male C57 BL/6 mice exposed for 5 consecutive days per week, to isoflurane (1.5% for 1 h/day) and administered with RSV (50 and 100 mg/kg for 35 days), demonstrated how this polyphenol, with its potent antioxidant properties, reduced the isoflurane reproductive toxicity by inhibiting free radicals and LP and increasing the antioxidant capacity in testicular tissue [53].

Antineoplastic agents cause oxidative damage and can alter the structure of organs, sexual hormones, and their function [213]. Among these, MTX, a folic acid antagonist with antiproliferative, anti-inflammatory, and immunomodulatory actions, causes functional abnormalities in both somatic and reproductive cells due to its inhibitory effect on DNA synthesis, repair, and cellular replication. Furthermore, at the testicular level, by increasing the ROS generation beyond a threshold level, it compromises spermatogenesis [213]. Sarman and co-workers revealed that RSV has positive effects on MTX-induced acute testicular damage, OS, and apoptosis in male Wistar albino rats [180]. The authors observed that the group of rats that were given RSV (20 mg/kg/day for 5 days) in combination with MTX showed significant increase in TAS and decrease in both OSI and TOS compared to the group treated with MTX alone. Moreover, the testicular tissue from MTX + RSV group also showed a negative immunoreactivity for proapoptotic markers caspase 3, 8, and 9 [180]. Many agents whose primary target is DNA are used as cancer chemotherapeutics. Among these, CIS is used against a variety of neoplasms, but like many chemotherapeutic drugs, it presents a range of side effects that limit its clinical application [214]. It alters male reproductive function in both humans and animals by causing severe damage to the testicles including germ cell apoptosis and OS, Leydig cell dysfunction, and testicular steroidogenic disorder, culminating in infertility [215]. A study conducted in male albino mice investigated the role of RSV against CIS-induced testicular damage [216]. In particular, when CIS was used in combination with RSV (1 mg/kg/day for 4 weeks), it not only determined an improvement in spermatic parameters such as sperm motility and concentration, but also reduced OS as evidenced by the increase in tissue expression levels of GSH, GPx, GST, SOD, and CAT and by the reduction in those of LP and NO [216]. Another in vivo study performed in male Wistar rats confirmed the ability of RSV to protect against testicular damage and reproductive dysfunction induced by CIS [181]. In fact, in the animals group to which RSV (20 mg/kg daily for 45 days) was administered in combination with CIS, an improvement in serum levels of T, FSH, and LH was observed, as well as an increase in the SPZ number and motility, and apoptosis and ERS inhibition related to antioxidant potential increase [181]. In particular, the testes from CIS + RSV-treated rats presented a high expression of antioxidant enzymes, including SOD, CAT, and GPx, and GSH, together with a down-regulation of those of cleaved caspase 3, BAX, P53, pAKT/pbad, cleaved calpain 1/caspase 12, p-ERK1/2, and p-SAPK/p-JNK [181].

Germ cell survival and differentiation into spermatids depend on T which is transformed by 5-α reductase into DHT in the prostate [217]. It has been reported that the use of finasteride, a 5-α reductase inhibitor drug used in the management of nodular prostatic hyperplasia and male pattern baldness, correlated with alterations in seminal parameters, difficulties in fertilization, and sexual problems such as decreased libido and erectile dysfunction [218]. An in vivo study revealed that RSV, due to its antioxidant and anti-apoptotic effects, had a protective effect against histopathological alterations in the seminiferous tubules of adult male Wistar albino rats caused by finasteride [219]. In particular, the animals group administered finasteride in combination with RSV (20 mg/kg/day for 8 weeks) revealed a considerable decrease in MDA levels together with an increase in SOD, GPx, and CAT levels compared to the finasteride-treated group alone [219]. Preserving reproductive health in cancer survivors is an important goal in cancer treatment, as conventional cytotoxic therapies can cause irreversible damage to the reproductive system. In this context, although ICIs, including anti-PD-1, have become a standard therapeutic approach for several malignancies, the impact of ICIs on reproductive function and fertility is not well understood. Studies have suggested that RSV has the potential to act as NRF2 agonist to counteract reproductive toxicity induced by various diseases, drugs, and environmental toxins. Recently, an in vivo study conducted on adult male C57 BL6/J mice with B16 melanoma demonstrated that RSV could offer protection against testicular toxicity following anti-PD-1 treatment, through its antioxidant and anti-ferroptosis properties [220]. In particular, it has been observed that anti-PD-1 treatment resulted in a marked reduction in sperm concentration, alterations in gonadal hormone levels and alterations in BTB integrity. Furthermore, in the testes of anti-PD-1 treated mice, the induction of OS was observed, which in turn triggered testicular ferroptosis together with increased T-cell infiltration and inflammatory cytokine expression. RSV supplementation (40 mg/kg) orally every other day for 1 month alleviates anti-PD-1-induced testicular toxicity by reducing T-cell infiltration, interferon-gamma levels, and activating the NRF2/SLC7 A11/GPX4 pathway that contributes to maintaining iron and lipid homeostasis [220].

The effects of RSV use on testicular function were evaluated and compared with that of the powerful and well-known antioxidant hydrogen sulfide SG1002 [221]. Both were administered for 75 days in subjects (54 men) affected by oligoasthenozoospermia to determine whether they could benefit from them. However, although the results of this clinical trial demonstrated that both antioxidants are well tolerated by the human body without developing significant adverse effects at the doses used, they highlighted that SG1002 is much more powerful and effective than RSV [221]. In fact, compared to the placebo group, treatment with SG1002 led to an increase in sperm concentration and motility and to the recovery of mobile forms, which are parameters that did not significantly improve in the group treated with RSV alone [221]. Although the results of this study are not comparable to others discussed so far in proving the beneficial antioxidant action at testicular level, the observed good tolerability and limitation of side effects are sufficiently promising to motivate further clinical studies on this natural compound.

Most in vivo studies on animal models (rats and/or mice) evaluating the RSV antioxidant effects on the testis used 20–50 mg/kg dosages; even if these concentrations are not generally achievable in humans through oral supplementation, such doses used in smaller animals are necessary to observe the biological effects, allowing the establishment of safety and toxicity profiles before moving on to human experimentation [222,223]. While the preclinical studies on RSV antioxidant potential related to testicular function improvement are substantial, similar investigations in humans are very poor. In general, human studies on RSV biological effects use a variety of doses, ranging from a few hundred milligrams to several grams/day. However, because RSV is extensively metabolized and rapidly eliminated, it shows a poor bioavailability, resulting in very little of the ingested dose reaching the systemic circulation [222]. Several strategies (i.e., RSV nanoencapsulation in lipid nanocarriers or liposomes, nanoemulsions, micelles, and insertion into polymeric particles, solid dispersions, and nanocrystals) are being explored to improve pharmacokinetic characteristics and thus beneficial effects of RSV [43]. Although several in vitro and some in vivo studies suggested that all these approaches have the potential to improve RSV bioavailability, supporting its clinical utility, many challenges remain in order to confirm RSV as an effective therapeutic agent.

**Table 2:** Antioxidant effects of RSV in the testis: in vivo studies. The symbols indicate increase (↑) or decrease (↓).

Animal Model	Animal Health Status	Testicular Toxicity by Compounds and/or Drugs	RSV Dose/Duration	Antioxidant Mechanism	Reference
Breeding boars	normal		20 mg/kg/day for 14 days	SOD ↑ MDA ↓	[199]
Adult Wistar rats	diabetic		50 mg/kg/day for 4 weeks	H_2_O_2_ ↓ SOD, CAT, GPx, GSH ↑	[182]
Wistar rats	diabetic		150 mg/kg/day from 33 to 110 days postpartum	MDA ↓ LP ↓	[201]
Adult rats	diabetic		150 mg/kg/day for 21 days	SOD, CAT, GPx ↑ MDA ↓	[202]
Wistar albino rats	normal	glyphosate	20 mg/kg/day for 8 weeks	MDA ↓ GSH, SOD ↑	[204]
Wistar rats	normal	malathion	2, 8, 20 mg/kg/day for 65 days	TAC ↑ LP ↓ MDA ↓	[205]
Adult Wistar Rats	normal	PVC	20 mg/kg/day for 60 days	SOD, CAT ↑ LP ↓	[206]
Adult Sprague Dawley rats	normal	sulfoxaflor	20 mg/kg/day for 28 days	MDA, GSSG, NO ↓ GSH ↑	[179]
Adult Wistar albino rats	normal	Fe_2_O_3_-NP	20 mg/kg/day for 8 weeks	SOD, CAT, GPx, GSH ↑ MDA ↓	[208]
BALB/c mice	normal	morphine	2, 8, 20 mg/kg/day for 2 weeks	NO ↓	[211]
Adult C57 BL/6 mice	normal	isoflurane	50 and 100 mg/kg/day for 35 days	LP ↓ TAC ↑	[53]
Wistar albino rats	normal	metotrexate	20 mg/kg/day for 5 days	TAS ↑ OSI, TOS ↓	[180]
Albino mice	normal	cisplatin	1 mg/kg/day for 4 weeks	GSH, GPx, GST, SOD, CAT ↑ LP ↓ NO ↓	[216]
Wistar Rats	normal	cisplatin	20 mg/kg/day for 45 days	SOD, CAT, GPx, GSH ↑	[181]
Adult Wistar albino rats	normal	finasteride	20 mg/kg/day for 8 weeks	MDA ↓ SOD, GPx, CAT ↑	[219]
Adult C57 BL6/J mice	B16 melanoma	anti-PD-1	40 mg/kg/alternate day for 1 month	NRF2/SLC7 A11/GPX4 pathway ↑ LP ↓ ferroptosis ↓	[220]

## 6. Conclusions

The studies discussed have highlighted that understanding the mechanisms underlying the ROS-dependent alteration of male reproductive function and the protective actions of natural polyphenols can provide useful information on both etiology and management of male infertility.

Although small quantities of ROS regulate SSCs self-renewal and SPZ capacitation, hyperactivation, AR, and sperm–oocyte fusion, high levels of ROS can cause OS damaging germ cells at various stages of development, particularly primary and secondary SPT and SPZ. Natural antioxidants play a protective role against OS-induced testicular damage, and here the antioxidant properties of RSV have been extensively studied and described. In vitro studies have indeed revealed that in the testis RSV protection from the deleterious effects of OS, may not only be due to its direct free radical scavenger action but also due to enhanced expression of genes involved in mitochondrial energy biogenesis, mainly through the SIRT1, NRF2, and HO-1 signaling pathway. Furthermore, several in vivo studies on animal models have confirmed that RSV administration induced protective effects on OS-dependent impaired sperm function during several chronic diseases, such as diabetes, or caused by gonadotoxic agents, including antineoplastic agents or environmental pollutants acting as endocrine disruptors.

Future studies are needed to clarify the precise molecular mechanisms of the protective antioxidant action of RSV at the testicular level and evaluate its clinical efficacy, especially in alleviating reproductive toxicity caused by various compounds able to generate OS. In particular, human clinical trials will be critical to help identify novel therapeutic targets and strategies useful for preserving and improving sperm quality. Given the poor bioavailability of RSV in vivo, clinical studies should include those aimed to evaluate the effects of standardized RSV formulations with high bioavailability; furthermore, they should be aimed at populations of patients with consolidated OS in order to verify how the use of such formulations can improve not only sperm parameters but also pregnancy rates and live births percentage. Future clinical studies should also take into account that chronic pathological conditions such as diabetes, aging, and tumors can influence ROS signaling and therefore also sperm function.

## Data Availability

Not applicable.

## Abbreviations

17β-HSD	17 beta-hydroxysteroid dehydrogenase
^1^O_2_	singlet oxygen
3β-HSD	3 beta-hydroxysteroid dehydrogenase
4-HNE	4-hydroxynonenal
8-OHdG	8-hydroxi-2′-deoxyguanosine
AIFM1	apoptosis-inducing factor 1, mitochondrial isoform X2
AKT	protein-kinase B
AMPK	adenosine monophosphate-activated protein kinase
AR	acrosome reaction
ARE	antioxidant response element
ASC	apoptosis associated speck-like protein
ASK1	apoptosis signal-regulating kinase 1
ATP	adenosine triphosphate
AZS	asthenozoospermia
B(a)P	benzo(a)pyrene
BAD	BCL2 associated agonist of cell death
BAK	Bcl-2 homologous antagonist killer
BAX	BCL2 associated x, apoptosis regulator
BCL2	B-cell lymphoma-2 protein
BCL6 B	B-cell CLL/lymphoma 6 member B protein
Bcl-xL	B-cell lymphoma-extra large
BTB	blood-testis barrier
cAMP	cyclic adenosine monophosphate
CAs	carbonic anhydrases
CAT	catalase
cGMP	cyclic guanosine monophosphate
CIS	cisplatin
CYTc	cytochrome c
CO_2_	carbon monoxide
CRISPR	clustered regularly interspaced short palindromic repeats
DAG	diacylglycerol
DAX1	dosage sensitive sex reversal (DSS), adrenal hypoplasia congenita (AHC) critical region on the X chromosome, gene 1
DEHP	diethylhexylphthalate
DHT	dihydrotestosterone
DISC	death-inducing signaling complex
EGG	ovum cell
ERK	extracellular signal-regulated kinase
ERS	endoplasmic reticulum stress
ETC	mitochondrial electron transport chain
FADD	Fas-associated death domain protein
FAS	fas cell surface death receptor
FGF2	fibroblast growth factor 2
FOXO1	forkhead box O protein 1
FOXOs	forkhead box O proteins
FSH	follicle-stimulating hormone
GJIC	gap junction intercellular communication
GLF	glyphosate
GDNF	neurotrophic factor derived from the glial cell line
GnRH	gonadotropin-releasing hormone
GPx	glutathione peroxidase
GPX1	glutathione peroxidase 1
GPX4	glutathione peroxidase 4
GSH	glutathione
GSSG	glutathione disulfide
GSS	glutathione synthetase
GST	glutathione-S-transferase
H^+^	hydrogen ion
H_2_O_2_	hydrogen peroxide
hCG	human chorionic gonadotropin
HCO_3_^−^	hydrogen carbonate ion
HO-1	heme oxygenase-1
Hx/XO	hypoxanthine/xanthine oxidase
ICIs	immune checkpoint inhibitors
IL-10	interleukin-10
IL-4	interleukin-4
IL-6	interleukin-6
JNK	c-jun n-terminal kinases
KEAP1	Kelch-like ECH-associated protein 1
LH	luteinizing hormone
LP	lipid peroxidation
MAPKs	mitogen-activated protein kinases
MDA	malondialdehyde
MMP	mitochondrial membrane potential
mTOR	mechanistic target of rapamycin
MTX	methotrexate
NADPH	nicotinamide adenine dinucleotide phosphate
NDUFA9	NADH dehydrogenase [ubiquinone] 1 alpha subcomplex subunit 9
NDUFS2	NADH dehydrogenase [ubiquinone] iron-sulfur protein 2
NDUFS8	NADH dehydrogenase [ubiquinone] iron-sulfur protein 8
SDHB	succinate dehydrogenase [ubiquinone] iron-sulfur subunit
NFkB	nuclear factor kappa B
NLRP3	nucleotide oligomerization domain (NOD)-like receptor family pyrin domain containing 3
NO	nitric oxide
NOS	nitric oxide synthase 1
NOX1	NADPH oxidase 1
NOX5	NADPH oxidase 5
NQO1	NADPH-quinone oxidoreductase 1
NR5A1	nuclear receptor subfamily 5 group A member 1
NRF2	nuclear factor-erythroid 2-related factor 2
O_2_	oxygen
O_2_^•−^	superoxide anion radical
OGG1	8-oxoguanine DNA glycosylase
OH	hydroxy group
OH^•^	hydroxyl radical
ONOO^−^	peroxynitrite
OS	oxidative stress
OSI	oxidative stress index
PAHs	polycyclic aromatic hydrocarbons
PARP-1	poly(ADP-ribose) polymerase 1
PD-1	programmed cell death protein 1
pERK1/2	extracellular signal-regulated kinase 1/2
PGC-1α	peroxisome proliferator-activated receptor-gamma coactivator 1-alpha
PI3K	fosfoinositide-3-kinasi
PIP2	phosphatidylinositol-4,5-bisphosphate
p-JNK	p-jun n terminal kinase
PKA	protein kinase A
PKC	protein kinase C
PKG	protein kinase G
PLA2	phospholipase A2
PMN	polymorphonuclear leukocytes
Prx4	peroxiredoxin 4
p-SAPK	p-stress-activated protein kinase/jun-amino-terminal kinase
PTEN	phosphatase and tensin homolog
PTK	protein tyrosine kinase
PTPase	phosphotyrosine phosphatase
PUFAs	polyunsaturated fatty acids
PVC	polyvinyl chloride
RNS	reactive nitrogen species
ROS	reactive oxygen species
RS	round spermatids
RSV	resveratrol
RTK	receptor tyrosine kinase
sAC	soluble adenylyl cyclase
SC	Sertoli cells
SF1	steroidogenic factor 1
SFK	Src family kinase
SIRT1	sirtuin 1
SLC7A11	solute carrier family 7 member 11
SOD	superoxide dismutase
SPG	spermatogonia
SPT	spermatocytes
SPZ	spermatozoa
SRC	proto-oncogene tyrosine-protein kinase Src
Srx	sulforidoxins
SSCs	spermatogonial stem cells
StAR	steroidogenic acute regulatory protein
STZ	streptozotocin
T	testosterone
TAC	total antioxidant capacity
TAS	total antioxidant status
TMEM225	transmembrane protein 225
TERT	telomerase reverse transcriptase
TFEB	transcription factor EB
TNF-α	tumor necrosis factor-alpha
TNF-R1	tumor necrosis factor receptor 1
TOS	total oxidant status
TRAIL-R1/2	tumor necrosis factor-related apoptosis-inducing ligand receptor 1/2
Trx	thioredoxin
ZEN	zearalenone
